# Exploring the effect of carbon nanoparticle tracing technique on five-year overall survival and disease-free survival in patients undergoing radical surgery for colorectal cancer: a retrospective study

**DOI:** 10.3389/fonc.2024.1514175

**Published:** 2024-12-18

**Authors:** Guangxu Wen, Zihao Jia, Yingying Wang, Qingjie Kang, Denghua Hu, Ziwei Wang

**Affiliations:** ^1^ Department of Gastrointestinal Surgery, The First Affiliated Hospital of Chongqing Medical University, Chongqing, China; ^2^ Neonatal Intensive Care Unit, Shandong Provincial Hospital, Jinan, Shandong, China

**Keywords:** carbon nanoparticle tracing technique, colorectal cancer, overall survival, disease-free survival, lymph node

## Abstract

**Background:**

To investigate the effect of preoperative carbon nanoparticle tracing technique via colonoscopy on the five-year overall survival and disease-free survival rates in patients undergoing radical resection for colorectal cancer.

**Methods:**

A retrospective cohort study was conducted to collect data from patients diagnosed with colorectal cancer who underwent radical resection with complete postoperative pathological information at the First Affiliated Hospital of Chongqing Medical University from March 2013 to February 2017. Patients with multiple primary cancers were excluded, resulting in 2,237 eligible patients in the study. Of these, 368 patients were lost to follow-up within five years after surgery, resulting in a final sample of 1,869 patients. These patients were then divided into two groups: 758 patients who underwent preoperative carbon nanoparticle tracing technique via colonoscopy (CAS group) and 1,111 patients who did not undergo carbon nanoparticle tracing (non-CAS group). Survival curves for both overall survival and disease-free survival were plotted for both groups based on follow-up results. Univariate and multivariate analyses were performed to investigate the effect of carbon nanoparticle tracing technique on the 5-year overall survival and disease-free survival rates in patients, as well as to explore the factors influencing these outcomes.

**Results:**

The results showed that the total number of lymph nodes detected in the tracing group 15(11,19) was significantly higher than that in the non-tracing group 11(7,15), with a statistically significant difference (p<0.05). The 5-year overall survival rates were 90.8% in the CAS group and 87.4% in the non-CAS group, and, while the disease-free survival rate were 88.5% and 83.4%, respectively. However, the differences between 5-year overall survival and disease-free survival between the two groups were not statistically significant (p>0.05). Both univariate and multivariate cox regression analyses demonstrated that patient age, tumor stage, postoperative chemoradiotherapy, postoperative radiotherapy, and postoperative tumor recurrence were independent factors influencing the 5-year overall survival and disease-free survival rates in colorectal cancer patients.

**Conclusion:**

Carbon nanoparticle tracing technique can effectively increase the total detected number of lymph nodes in patients with radical resection for colorectal cancer, but it does not significantly impact the 5-year overall survival and disease-free survival rates in these patients.

## Introduction

Colorectal cancer is a common malignant tumor of the digestive tract. Studies have shown that colorectal cancer is currently the third most prevalent type of cancer worldwide (1, 2). In 2020, over 1.93 million cases of colorectal cancer were diagnosed globally, accounting for 10% of all cancer diagnoses, with more than 930,000 deaths attributed to the disease. In 2022, colorectal cancer ranked second in the estimated incidence of cancer in China and fifth in mortality rates (3). The incidence of colon cancer is increasing, with most patients being diagnosed at an advanced stage. Some scholars predict that by 2025, the number of colorectal cancer cases and deaths in China will reach approximately 6.423 million and 2.211 million, respectively (4). Despite continuous advancements in medical technology and treatment methods, nearly 60% of the cancer patients still fail to achieve a “clinical cure” (5, 6).

Recurrence and metastasis are crucial factors hindering clinical cure of colorectal cancer, with lymph node metastasis being one of the primary causes. Accurate preoperative assessment of lymph node metastasis, thorough intraoperative dissection of potentially metastatic lymph nodes, and maximizing the number of lymph nodes retrieved are all of great significance in accurately assessing pathological staging, guiding postoperative adjuvant treatment plans, and improving survival rates (7–10).

To maximize the number of lymph nodes retrieved, various lymph node tracing techniques have been developed, such as India ink, indocyanine green fluorescence imaging, and dye tracing methods (methylene blue, toluidine blue, etc.). However, each of these methods has its inevitable drawbacks. In recent years, carbon nanoparticle tracing technique has gradually attracted attention from scholars. Carbon nanoparticles (CNs) have a diameter of approximately 150 to 200 nm, which lies between that of capillaries (5 to 6 nm) and lymphatic vessels (about 500 nm). Therefore, after CNs are injected into the intestinal mucosa surrounding tumor tissues, they can only enter the lymphatic vessels and not the capillaries, allowing for accurate localization of lymph nodes. Multiple studies have demonstrated that CN tracing technique can significantly increase the number of lymph nodes detected during radical resection surgeries for colorectal (11–13), breast (14, 15), and gastric cancers (16, 17), and it also exhibits high safety and lymphatic tropism (18, 19). However, there have been no reports on whether CN tracing technique can improve patients’ five-year overall survival and disease-free survival rates. Therefore, data from 1,869 patients who underwent radical resection for colorectal cancer at the First Affiliated Hospital of Chongqing Medical University from March 2013 to February 2017 were collected in this study. Based on follow-up results, survival curves for patients’ five-year overall survival and disease-free survival were plotted, and univariate and multivariate analyses were conducted to explore the effect of CN tracing technique on these outcomes.

## Patients and methods

### Patients

In this retrospective cohort study, clinical data from 2,237 patients who underwent laparoscopic radical resection for colorectal cancer at the First Affiliated Hospital of Chongqing Medical University between March 2013 and February 2017 were included. Among them, 368 patients were lost to follow-up by the fifth postoperative year, leaving a total of 1,869 patients for this study. These patients were then divided into two groups based on whether preoperative endoscopic carbon nanoparticle suspension was used for lesion localization: the localization group with 758 patients and the non-localization group with 1,111 patients. Specific information on the patients can be found in **Table 1**.

**Table 1:** Clinical characteristics of patients.

Characteristics	Number(1869)
Age, year	64(55,72)
Sex	
Male Female	1088 (58.2%) 781(41.8%)
Tumor location	
Colon	791 (42.3%)
Rectum	1078 (57.7%)
Tumor size, cm	4.0(3.0,5.0)
Tumor stage	
1	355 (19.0%)
2	697 (37.3%)
3	727(38.9%)
4	90 (4.8%)
Surgical site	
Rectal resection	1107(59.23%)
Sigmoid resection	192(10.27%)
Left-side hemicolectomy	168(8.99%)
Right-side hemicolectomy	359(19.21%)
Transverse colon resection	15(0.80%)
Primary focal + metastases resection	28(1.50%)
Number of lymph nodes	12(9,16)
Pre-operative chemotherapy	
Yes	190(10.2%)
No	1679(89.8%)
Pre-operative radiotherapy	
Yes	70 (3.7%)
No	1799(96.3%)
Post-operative chemotherapy	
Yes	1660(88.82%)
No	209(11.18%)
Post-operative radiotherapy	
Yes	9(0.48%)
No	1860(99.52%)
Post-operative Immunotherapy	
Yes	13(0.70%)
No	1856(99.30%)
Tumor recurrence	
Yes	236(12.63%)
No	1633(87.37%)
Patient death	
Yes	225(12.04%)
No	1644(87.96%)
DFS	60(60,60)
OS	60(60,60)
CAS	
Yes	758 (40.6%)
No	1111(59.4%)
Total lymph nodes	

Variables are expressed as the median(IQR), number (frequency %).

CAS, carbon nanoparticles staining.

### Inclusion and exclusion criteria

Inclusion Criteria: 1. Age: over 18 years; 2. Diagnosis: histologically confirmed colorectal cancer; 3. Treatment modality: radical resection surgery for colorectal cancer; 4. Intervention: preoperative carbon nanoparticle marking via colonoscopy.

Exclusion Criteria: 1. Patients who did not receive radical surgery (e.g., those who underwent local excision or received palliative treatment); 2. Multiple primary cancers; 3. Missing clinical data. 4. Complicated with severe cardiopulmonary, hepatic and renal disease or coagulation disorders.

### Preoperative carbon nanoparticle injection for lesion localization

One to seven days before laparoscopic resection for colorectal cancer (excluding neoadjuvant therapy), 4-6 points were marked with carbon nanoparticle suspension around the inner wall of the intestinal lumen at 1-2 cm from the anal side of the lesion, with approximately 1ml at each point. For small lesions, the injection was performed submucosally near the base of the lesion using the same method. If the base was small, an additional injection point was added in the submucosa area on the opposite side of the tumor.

### Surgical methods

All patients strictly underwent surgery in accordance with complete mesocolic excision (CME) and total mesorectal excision (TME). Regional lymph node dissection for colon cancer must encompass paracolic, mesenteric and central lymph nodes. In this study, regional lymph node dissection was performed on all patients using D3 radical surgery, mainly including the resection of black-stained lymph nodes, the removal of lymph nodes based on the course of blood vessels, lymph nodes palpable by hand, and lymph nodes that were black-stained and visible outside the resection range of the radical procedure.

### Postoperative evaluation and follow-up

Patients were followed up through a combination of telephone calls and outpatient visits. Follow-ups were conducted every 3-6 months within the first two years after surgery and annually thereafter. Tumor recurrence and patient survival status within 5 years were investigated during the follow-up. Based on the follow-up results, overall survival and disease-free survival curves were plotted, and the overall survival and disease-free survival rates for patients who received preoperative carbon nanoparticle tracing technique were calculated.

### Statistical analysis

Continuous variables that did not follow normal distribution were expressed as the median (Interquartile Range (IQR)) and categorical variables were presented as number and frequency (%). Differences between groups were compared by the Mann-Whitney U test and Kruskal-Wallis test for continuous variables and the chi-square test or Fisher’s exact test for categorical variables. Univariate and multivariate cox regression models were used to evaluate the association between the number of lymph nodes harvested and the survival rate. Survival curves were obtained using the Kaplan-Meier method and the Log-rank test was used for comparison of the groups. P<0.05 was considered statistically significant. All statistical analyses were performed with SPSS for Windows (version 25.0, SPSS Inc).

## Results

### Patient characteristics

From March 2013 to February 2017, a total of 2,237 patients were enrolled in this study, of which 368 patients were excluded due to loss to follow-up, leaving 1,869 patients for inclusion. There were 1,088 males and 781 females, with a median age 64 years. Among them, 758 patients received a preoperative carbon nanoparticle injection, while 1,111 patients did not. As shown in **Table 1**. The number of lymph nodes retrieved in the CAS group (15(11,19)) was significantly higher than that in the non-CAS group (11(7,15))(*p*< 0.05) as shown in **Table 2**.

**Table 2:** Comparison between the CAS group and Non-CAS group.

Characteristics	CAS (758)	Non-CAS (1111)	P value
Age, year	64 (53,71)	64 (57,73)	0.167
Sex			0.757
Male Female	445 (58.7%) 313 (41.3%)	643 (57.9%) 468 (42.1%)	
Tumor location			<0.001*
Rectum	385 (50.8%)	693 (62.4%)	
Colon	373 (49.2%)	418 (37.6%)	
Tumor size	4.0 (3.0,5.0)	4.0 (3.0,5.0)	0.222
Tumor stage			0.023*
1	161 (21.3%%)	194 (17.5%)	
2	288 (38.0%)	408 (36.7%)	
3	282 (37.3%)	445 (40.0%)	
4	26 (3.4%)	64 (5.8%)	
Surgical site			<0.001*
Rectal resection	398 (52.5%)	709 (63.8%)	
Sigmoid resection	103 (13.6%)	89 (8.0%)	
Left-side hemicolectomy	87 (11.5%)	81 (7.3%)	
Right-side hemicolectomy	152 (20.0%)	207 (18.6%)	
Transverse colon resection	10 (1.3%)	5 (0.5%)	
Primary focal + metastases resection	8 (1.1%)	20 (1.8%)	
The number of lymph nodes	15 (11,19)	11 (7,15)	<0.001*
Preoperative chemotherapy			0.239
No	689 (90.9%)	990 (89.1%)	
Yes	69 (9.1%)	121 (10.9%)	
Preoperative radiotherapy			0.014*
No	740 (97.6%)	1059 (95.3%)	
Yes	18 (2.4%)	52 (4.7%)	
Postoperative Chemotherapy			0.969
No	84 (11.1%)	125 (11.3%)	
Yes	674 (88.9%)	986 (88.7%)	
Postoperative radiotherapy			0.434
No	756 (99.7%)	1104 (99.4%)	
Yes	2 (0.3%)	7 (0.6%)	
Postoperative Immunotherapy			0.662
No	754 (99.5%)	1102 (99.2%)	
Yes	4 (0.5%)	9 (0.8%)	
Tumor recurrence			0.378
No	669 (88.3%)	964 (86.8%)	
Yes	89 (11.7%)	147 (13.2%)	
Patient death			0.492
No	672 (88.7%)	972 (87.5%)	
Yes	86 (11.3%)	139 (12.5%)	
DFS	60 (60,60)	60 (60,60)	0.308
OS	60 (60,60)	60 (60,60)	0.346

Variables are expressed as the median(IQR), number, frequency (%), *P-value <0.05.

CAS, carbon nanoparticles staining.

### Survival

**Figure 1:**
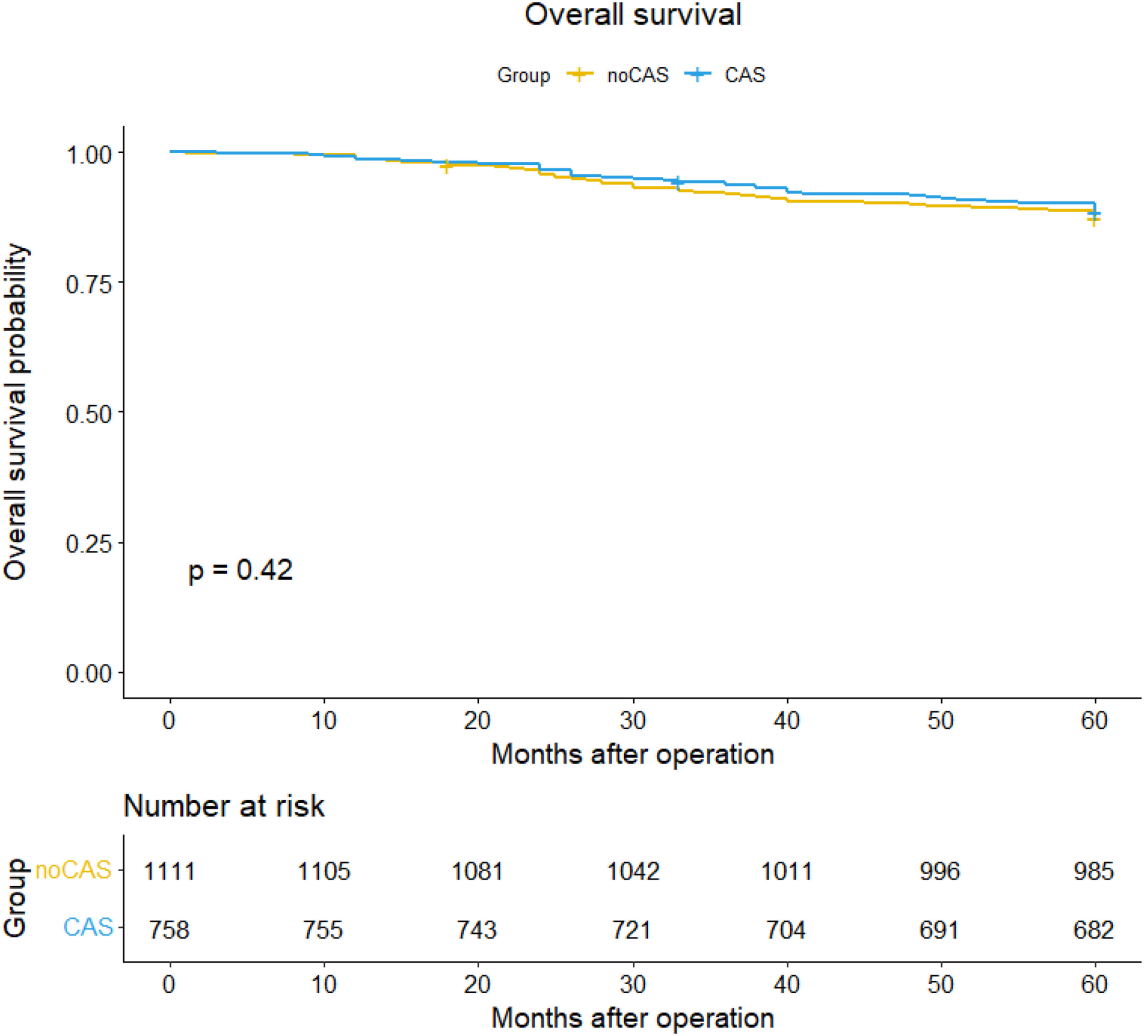
Overall survival curve and survival table.

**Figure 2:**
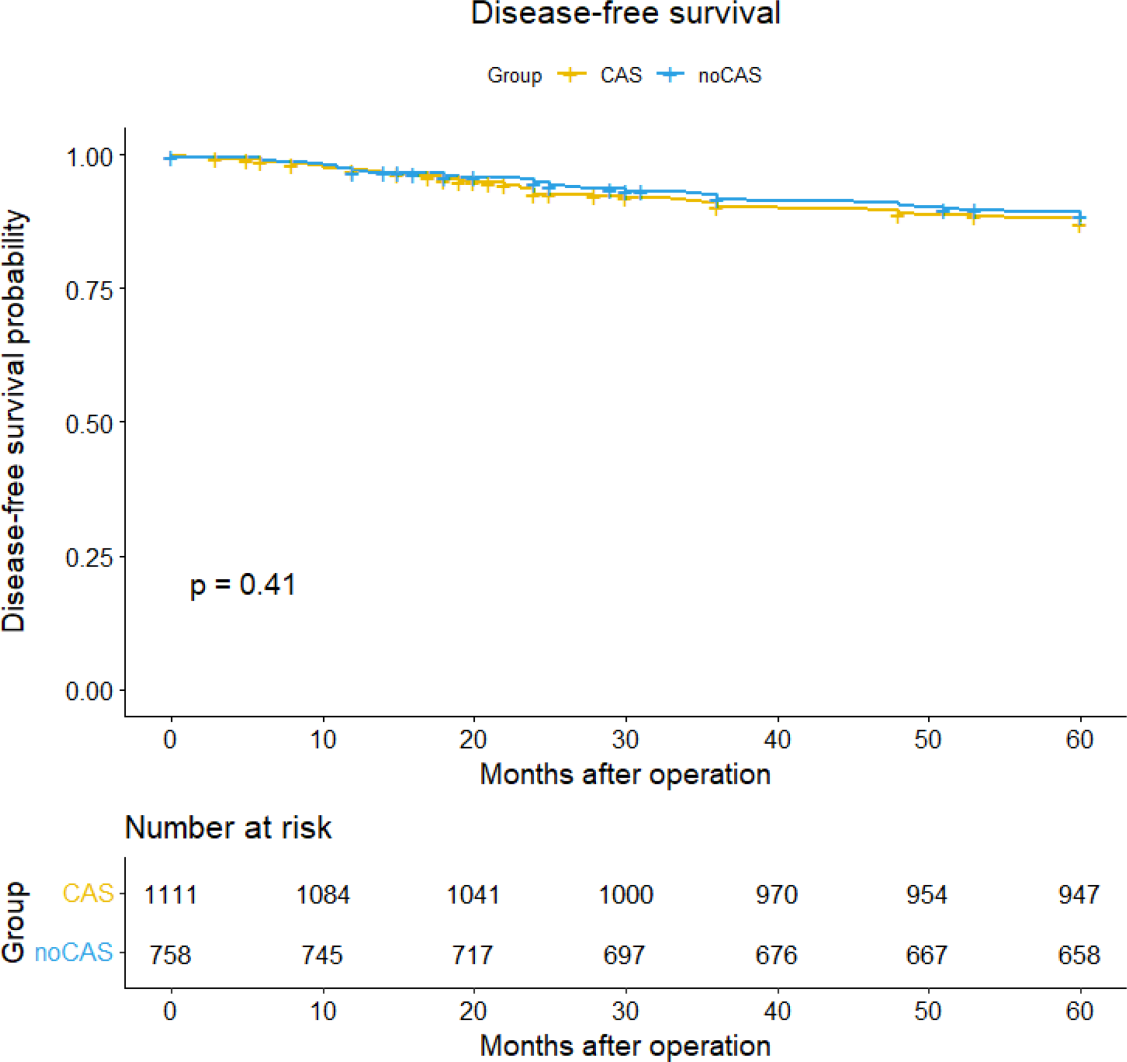
Disease-free survival curve and survival table.

Based on the patient data obtained from follow-ups, survival curves and survival tables for five-year overall survival and disease-free survival rates were plotted for the CAS and non-CAS groups, as shown in **Figures 1** and **2**. The study results revealed that the five-year overall survival rate were 90.8% for the CAS group and 87.4% for the non-CAS group, while the disease-free survival rate were 88.5% and 83.4% respectively. The Log rank test showed no statistically significant difference in five-year overall survival and disease-free survival rates between the CAS and Non-CAS groups (*p*=0.42, *p*=0.41, *p*> 0.05).

### Univariate and multivariate cox regression analysis of overall survival

As shown in **Table 3**, the result of univariate cox regression analysis showed that patient age, tumor stage, tumor size, surgical procedures, postoperative chemotherapy, postoperative radiotherapy and postoperative tumor recurrence were significantly influencing the overall survival of colorectal cancer patients (*p* < 0.05). The result of multivariate cox regression analysis showed that patient age, tumor stage, postoperative chemoradiotherapy, postoperative radiotherapy and postoperative tumor recurrence were independent factors influencing the overall survival rate in colorectal cancer patients (*p* < 0.05).

**Table 3:** Univariate and multivariate cox regression analysis of overall survival.

Risk factors	Univariate analysis		Multivariate analysis	
	HR (95% CI)	P value	HR (95% CI)	P value
Age (years)	1.052 (1.039-1.065)	<0.001*	1.038 (1.026-1.050)	<0.001*
Sex(male/female)	0.922 (0.706-1.204)	0.552		
Tumor location (colon/rectum)	1.282 (0.987-1.665)	0.061		
Tumor stage (4/3/2/1)	1.318 (1.122-1.548)	<0.001*	1.249 (1.043-1.495)	0.016*
Tumor size, cm	1.147 (1.088-1.209)	<0.001*		
Surgical procedures	1.115 (1.017-1.223)	0.021*		
Number of lymph nodes	1.007 (0.990-1.024)	0.416		
Preoperative chemotherapy	0.801 (0.501-1.281)	0.354		
Preoperative radiotherapy	1.194 (0.634-2.251)	0.583		
Postoperative chemotherapy	0.406 (0.300-0.556)	<0.001*	0.676 (0.489-0.935)	0.018*
Postoperative radiotherapy	4.998 (1.859-13.44)	0.001*	3.717 (1.364-10.131)	0.010*
Postoperative immunotherapy	1.301 (0.323-5.233)	0.711		
Tumor recurrence	43.77 (31.74-60.35)	<0.001*	39.94 (28.792-55.400)	<0.001*
CAS	0.423 (0.896-1.172)	0.412		

*P-value <0.05.

HR, hazard ratio; CI, confidence interval; CAS, carbon nanoparticles staining.

### Univariate and multivariate cox regression analysis of disease-free survival

As shown in **Table 4**, the result of univariate cox regression analysis showed that patient age, tumor stage, tumor size, surgical approach, postoperative chemotherapy, postoperative radiotherapy and postoperative tumor recurrence were significantly influencing overall survival of colorectal cancer patients (*p* < 0.05). The result of multivariate cox regression analysis showed that patient age, tumor stage, postoperative chemoradiotherapy, postoperative radiotherapy and postoperative tumor recurrence were independent factors influencing the disease-free survival rate in colorectal cancer patients (*p* < 0.05).

**Table 4:** Univariate and multivariate cox regression analysis of disease-free survival.

Risk factors	Univariate analysis		Multivariate analysis	
	HR (95% CI)	P value	HR (95% CI)	P value
Age (years)	1.051 (1.038-1.064)	<0.001*	1.029 (1.016-1.041)	<0.001*
Sex(male/female)	0.911 (0.698-1.190)	0.496		
Tumor location (colon/rectum)	1.282(0.987-1.665)	0.063		
Tumor stage (4/3/2/1)	1.315(1.125-1.537)	<0.001*	1.245 (1.046-1.482)	0.014*
Tumor size, cm	1.148 (1.089-1.210)	<0.001*		
Surgical Procedures	1.118 (1.019-1.227)	0.018*		
Number of lymph nodes	1.007 (0.991-1.025)	0.391		
Preoperative chemotherapy	0.832 (0.520-1.210)	0.442		
Preoperative radiotherapy	1.285 (0.682-2.423)	0.438		
Postoperative chemotherapy	0.409 (0.300-0.560)	<0.001*	0.700 (0.506-0.967)	0.031*
Postoperative radiotherapy	4.956 (1.843-13.33)	<0.001*	2.901(1.064-7.912)	0.037*
Postoperative immunotherapy	1.398 (0.348-5.626)	0.637		
Tumor recurrence	82.43 (58.07-117)	<0.001*	74.313 (51.686-106.845)	<0.001*
CAS	0.894 (0.683-1.169)	0.412		

*P-value <0.05.

HR, hazard ratio; CI, confidence interval; CAS, carbon nanoparticles staining.

The number of lymph nodes detected between the three groups(stage III) was significantly different (P<0.05), among which stage IIIA was significantly different from stage IIIC (P=0.001) and stage IIIB was significantly different from stage IIIC (P=0.0003). The results were shown in **Table 5**.

**Table 5:** Number of lymph nodes detected in patients (stage III).

Characteristics	Number of lymph nodes	P value
Stage III		0.0002*
IIIA	11 (8,15)	
IIIB	12 (9,16)	
IIIC	14 (10,18)	

IIIA and IIIC:P=0.001; IIIB and IIIC: P=0.0003.

There were significant differences in the number of lymph nodes of the CAS group among the three stage (P=0.027). Among which stage IIIA was significantly different from stage IIIC (P=0.012).

There were significant differences in the number of lymph nodes of the Non-CAS group among the three stage(P=0.0002).Among which stage IIIA was significantly different from stage IIIB (P=0.0076),IIIA was significantly different from stage IIIC (P=0.0001) and IIIB was significantly different from stage IIIC (P=0.0135).The results were shown in **Table 6**.

**Table 6:** Number of lymph node detection in patients between CAS and non-CAS groups (III stage).

Characteristics	CAS	P value	Non-CAS	P value
Stage III		0.027*		0.0002*
IIIA	15 (12,17)		9.5 (6.5,11)	
IIIB	14.5 (10,18)		11 (8,14)	
IIIC	16 (13,21)		12 (9,16)	

Group CAS: IIIA and IIIC: P=0.012.

Group Non-CAS: IIIA and IIIB: P=0.0076; IIIA and IIIC: P=0.0001; IIIB and IIIC: P =0.0135.

The number of lymph nodes detected between the CAS and non-CAS groups of the three stage was significantly different s(P<0.05).The results were shown in **Table 7**.

**Table 7:** Number of total lymph nodes detected between the CAS and non-CAS groups (III stage).

Characteristics	CAS	Non-CAS	P value
IIIA	15 (12,17)	9.5 (6.5,11)	<0.0001*
IIIB	14.5 (10,18)	11 (8,14)	<0.0001*
IIIC	16 (13,21)	12 (9,16)	<0.0001*

*P-value<0.05

**Figure 3:**
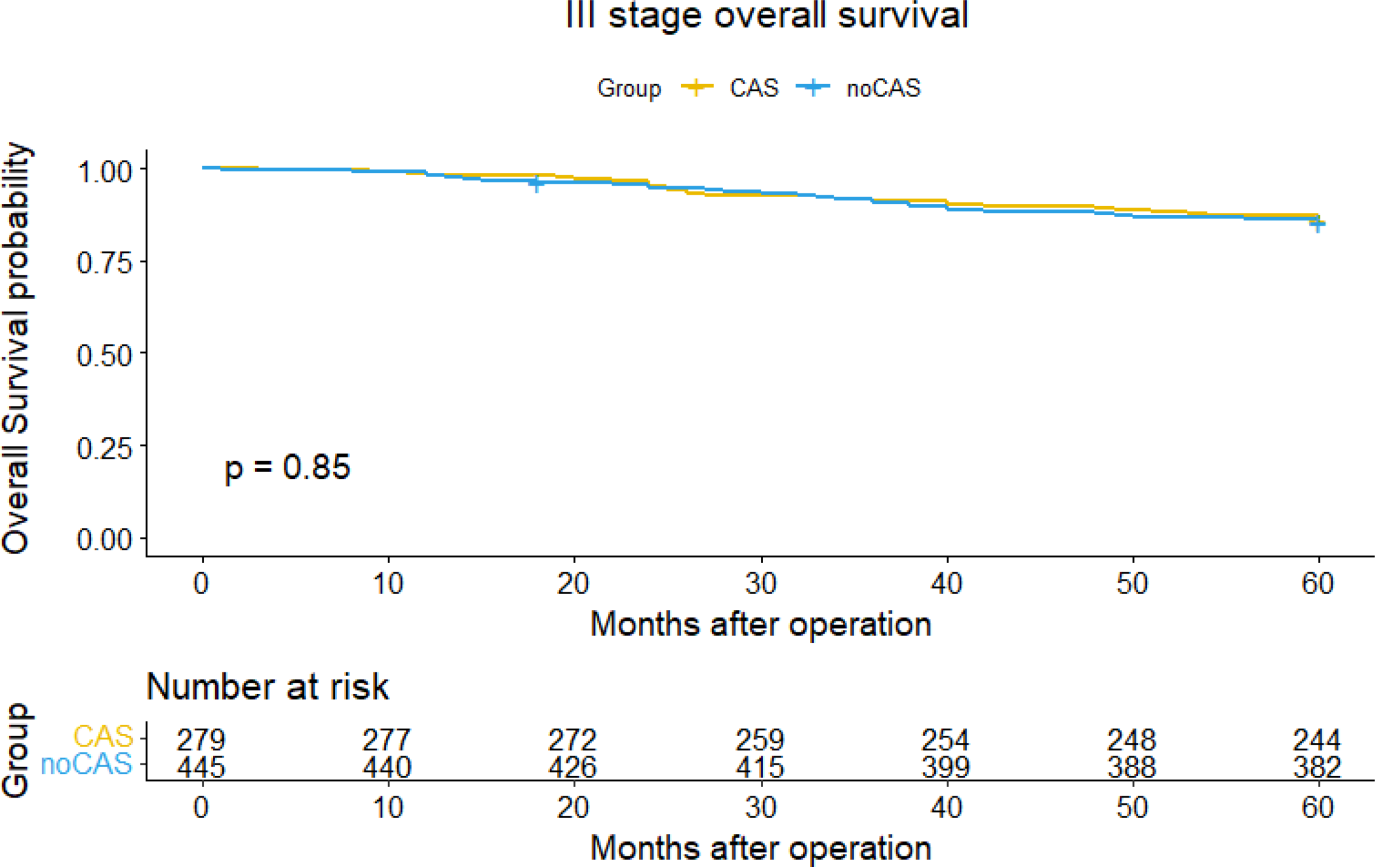
Overall survival curve and survival table (stage III).

**Figure 4:**
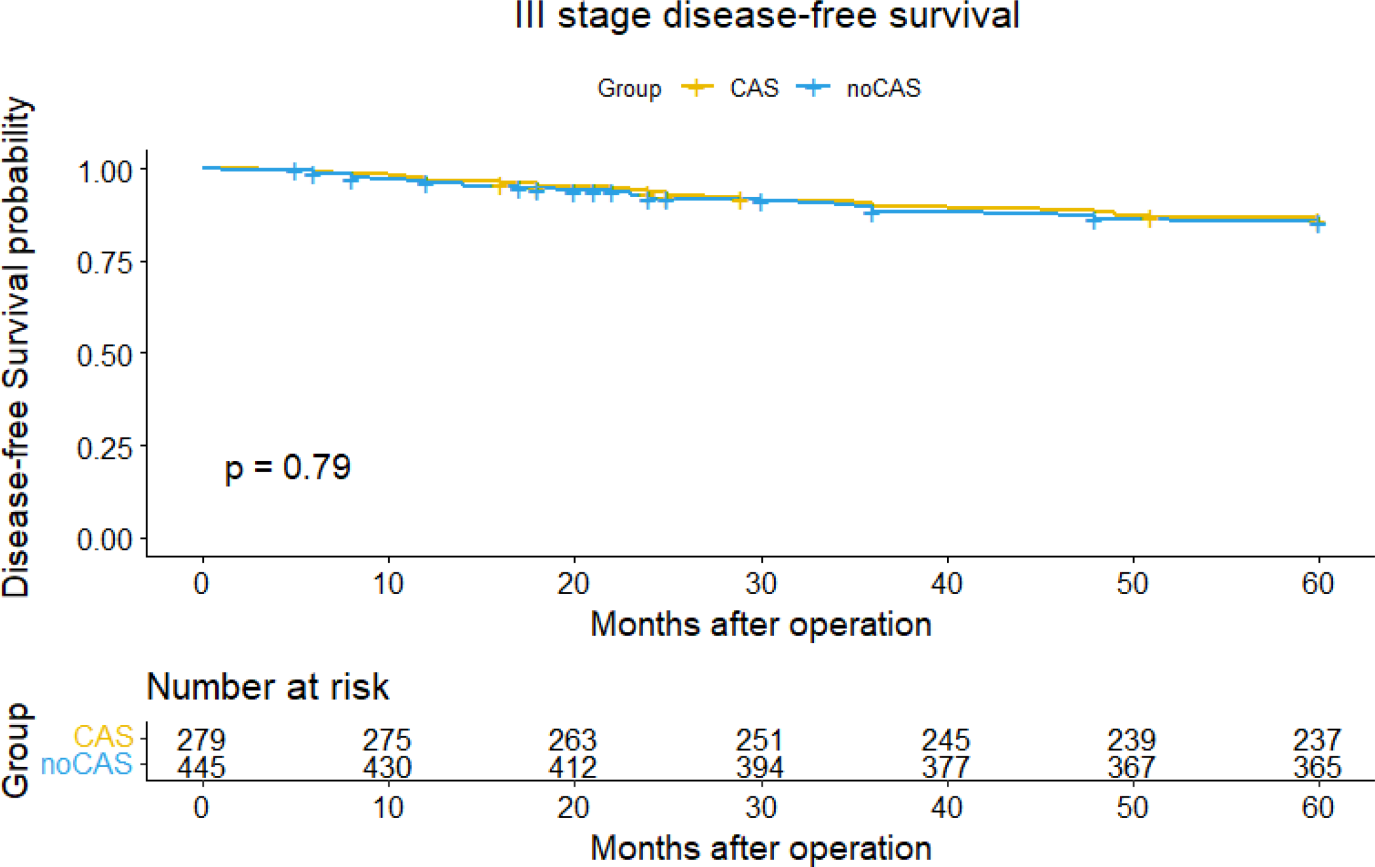
Disease-free survival curve and survival table (stage III).

The **Figures 3** and **4** showed that the difference of five-year overall survival and disease-free survival between the two groups was not statistically significant (P>0.05).

### Univariate and multivariate cox regression analysis of overall survival and disease-free survival of stage III

The result of univariate cox regression analysis showed that patient age, tumor size, postoperative chemotherapy, postoperative radiotherapy and postoperative tumor recurrence were significantly influencing overall survival and disease-free survival of colorectal cancer stage III patients (*p* < 0.05). The result of multivariate cox regression analysis showed that patient age, postoperative chemoradiotherapy, postoperative radiotherapy and postoperative tumor recurrence were independent factors influencing the overall survival rate of colorectal cancer stage III patients (*p* < 0.05). Multivariate cox regression analysis showed that postoperative chemoradiotherapy and tumor recurrence were independent factors influencing the disease-free survival rate of colorectal cancer stage III patients (*p* < 0.05). The results were shown in **Tables 8** and **9**.

**Table 8:** Univariate and multivariate cox regression analysis of overall survival rate (III stage).

Risk factors	Univariate analysis		Multivariate analysis	
	HR (95% CI)	P value	HR (95% CI)	P value
Age (years)	1.034 (1.016-1.052)	<0.001*	1.029 (1.011-1.047)	0.002*
Sex(male/female)	0.8442 (0.569-1.253)	0.400		
Tumor location (colon/rectum)	1.312 (0.891-1.932)	0.169		
Tumor stage (A/B/C)	1.208 (0.853-1.71)	0.287		
Tumor size, cm	1.181 (1.086-1.284)	<0.001*		
Surgical site (Rectal resection/Sigmoid resection/Left-side hemicolectomy/Right-side hemicolectomy/Transverse colon resection/Primary focal + metastases resection)	1.096 (0.941-1.277)	0.24		
Number of lymph nodes	0.998 (0.967-1.030)	0.887		
Preoperative chemotherapy(Yes/No)	0.532 (0.216-1.306)	0.168		
Preoperative radiotherapy(Yes/No)	0.552 (0.136-2.236)	0.405		
Postoperative chemotherapy(Yes/No)	0.228 (0.144-0.363)	<0.001*	0.450 (0.277-0.732)	0.001*
Postoperative radiotherapy(Yes/No)	9.504 (3.006-30.05)	0.0001*	3.296 (1.024-10.610)	0.046*
Postoperative immunotherapy(Yes/No)	0.731 (1.413-10.13)	0.711		
Tumor recurrence(Yes/No)	47.83 (28.82-79.38)	<0.001*	45.872 (27.277-77.143)	<0.001*
CAS(Yes/No)	0.963 (0.647-1.435)	0.854		

*P-value<0.05

**Table 9:** Univariate and multivariate cox regression analysis of disease-free survival rate (III stage).

Risk factors	Univariate analysis		Multivariate analysis	
	HR (95% CI)	P value	HR (95% CI)	P value
Age (years)	1.033 (1.015-1.050)	<0.001*		
Sex(male/female)	0.838 (0.564-1.243)	0.378		
Tumor location (colon/rectum)	1.317 (0.894-1.94)	0.163		
Tumor stage (A/B/C)	1.211 (0.856-1.714)	0.280		
Tumor size, cm	1.185 (1.089-1.288)	<0.001*		
Surgical site (Rectal resection/Sigmoid resection/Left-side hemicolectomy/Right-side hemicolectomy/Transverse colon resection/Primary focal + metastases resection)	1.101 (0.945-1.284)	0.217		
Number of lymph nodes	0.997 (0.966-1.030)	0.868		
Preoperative chemotherapy(Yes/No)	0.550 (0.224-1.351)	0.193		
Preoperative radiotherapy(Yes/No)	0.599 (0.148-2.429)	0.473		
Postoperative chemotherapy(Yes/No)	0.232 (0.146-0.369)	<0.001*	0.451 (0.278-0.732)	0.001*
Postoperative radiotherapy(Yes/No)	9.653 (3.055-30.49)	0.001*		
Postoperative immunotherapy(Yes/No)	1.624 (0.223-11.64)	0.630		
Tumor recurrence(Yes/No)	118.0 (63.59-219.0)	<0.001*	106.983 (56.991-200.829)	<0.001*
CAS(Yes/No)	0.948 (0.637-1.412)	0.794		

*P-value<0.05

In summary, our study demonstrates that the use of carbon nanoparticle tracing technique significantly increases the total number of lymph nodes retrieved in colorectal cancer patients but has no significant effect on their five-year overall survival or disease-free survival rates. However, patient age, tumor stage, postoperative chemoradiotherapy, and tumor recurrence have significant effects on both overall survival and disease-free survival rates. Patients with stage III colorectal cancer also showed the same results, and postoperative chemotherapy and tumor recurrence had a significant impact on both overall and disease-free survival in stage III patients.

## Discussion

Lymph node metastasis is a crucial pathway for the spread of colorectal cancer. Lymph node exploration allows for the identification of both the number and location of metastatic lymph nodes, enabling accurate staging of the cancer and providing essential basis for subsequent treatment. Moreover, the extent of lymph node metastasis is closely associated with patient prognosis. By assessing the extent of lymph node metastasis through lymph node exploration, physicians can evaluate patient prognosis and devise appropriate treatment plans. Additionally, the results of lymph node exploration can guide the selection of appropriate adjuvant therapies to reduce the risk of recurrence. Therefore, accurately dissecting lymph nodes and maximizing the number of lymph nodes retrieved are crucial in improving the cure rate in colorectal cancer patients (20–22).

In recent years, numerous lymph node tracing methods, such as India ink (23), methylene blue (24), and indocyanine green (ICG) fluorescence (25), have been applied in surgical procedures, each with their respective drawbacks. India ink suffers from poor diffusion properties, long retention time, and potential adverse reactions during marking, including inflammatory responses, local ulcers, and even obstructive necrosis (26). Methylene blue, on the other hand, has a small particle diameter and rapid diffusion which results in a short marking duration and can hinder surgical visibility. ICG fluorescence, which requires near-infrared light for visualization, significantly limits its widespread application (27). Recently, a novel carbon nanoparticle tracing technique has garnered substantial attention. Carbon nanoparticles (28), with diameters precisely between those of capillaries and capillary lymphatics, can easily enter capillary lymphatics but not capillaries, imparting excellent lymphatic system tropism and safety (29). Additionally, after local injection, carbon nanoparticles are phagocytosed by macrophages, allowing their entry into the lymphatic system and *in vivo* staining of lymph nodes in tumor regions. In a study by Renjie Wang et al. (19) involving 239 patients with stage I-III colorectal cancer who received the preoperative carbon nanoparticle tracing technique, no allergic reactions, drug-related complications, acute or chronic toxicity, or other adverse effects were observed. According to relevant studies, the carbon nanoparticle tracing technique has been extensively applied in radical resection for colorectal cancer. J W Cai et al. (31) found that among 1,421 patients undergoing radical resection for colorectal cancer, the total number of lymph nodes detected in the carbon nanoparticle tracing group was significantly higher than that in the control group (22.2 ± 11.2 vs. 19.0 ± 9.5, t=3.025, *p*=0.003), consistent with the conclusions of this study. A study by Rong Wang et al. (32) involving 113 patients with advanced colorectal cancer revealed that the carbon nanoparticle marking group reduced intraoperative lesion exploration time and total surgery duration, with less intraoperative blood loss and a relatively higher anus-preserving rate. However, the effect of the carbon nanoparticle tracing technique on the five-year overall survival rate and disease-free survival rate in colorectal cancer patients remains unreported.

This study aims to collect a substantial sample of clinical data from patients to more accurately and reliably analyze the effect of carbon nanoparticle tracing technique on the five-year overall survival rate and disease-free survival rate in patients undergoing radical resection for colorectal cancer. Our findings revealed that the number of lymph nodes detected in the carbon nanoparticle tracing group was significantly higher than that in the control group (localized group: 15.6 ± 7.4, non-localized group: 11.5 ± 6.5). This result is consistent with the results in a previous report by Yang Bin et al., who found that the lymph node staining rate after carbon nanoparticle injection was 56.8% (412/725), and that carbon nanoparticle lymphatic tracing could increase the number of lymph nodes dissected, particularly lymph nodes with diameters <5mm [4.6% (33/725)], which was significantly higher than in the non-tracing group [2.0% (10/478), *p*=0.025]. Similarly, Xiangchun Zhang et al. studied the medical records of 53 patients undergoing laparoscopic radical resection for colorectal cancer and found that the average number of lymph nodes detected and the number of micro-lymph nodes (<5mm) detected in the carbon nanoparticle injection group were significantly higher than those in the unstained control group [(16.7 ± 3.2) vs. (12.6 ± 2.3), *p*<0.01; (5.3 ± 2.4) vs. (2.1 ± 1.2), *p*<0.01].

Furthermore, by calculating the five-year overall survival rate, disease-free survival rate, and their survival curves for the localization group and the non-localization group, we found that carbon nanoparticle tracing technique had no significant effect on the five-year overall survival rate or disease-free survival rate of patients. This is consistent with the findings of a report by Liyu Wang et al. (30) who observed no significant difference in the 3-year survival rate between the carbon nanoparticle tracing group and the control group across stages I-IV of colorectal cancer. We attribute this to the multitude of factors influencing the long-term prognosis of colorectal cancer, such as patient age, clinical symptoms and complications, primary tumor location, postoperative adjuvant therapy, histological type and differentiation of the tumor, tumor recurrence, lymph node metastasis, and depth of invasion. The application of carbon nanoparticle tracing technique alone can only effectively increase the number of lymph nodes detected to a certain extent and improve the reliability of postoperative pathological N staging, but its effect on the five-year survival rate is limited.

To further identify the factors influencing the five-year overall survival rate and disease-free survival rate of patients, we conducted both univariate and multivariate analyses. The results indicated that patient age, tumor stage, postoperative radiotherapy and chemotherapy, as well as the postoperative recurrence status of the patients, were independent factors affecting the overall survival rate of colorectal cancer patients. Similarly, patient age, tumor stage, postoperative radiotherapy and chemotherapy, and tumor recurrence status were identified as independent factors influencing the disease-free survival rate. However, the use of preoperative carbon nanoparticle tracing technique did not affect the five-year overall survival rate or disease-free survival rate in the patients.

Finally, focusing on data from patients with stage III colorectal cancer, we found that nanocarbon tracer technology increased the number of lymph nodes detected in patients with stage III colorectal cancer, but did not affect the patients’ 5-year overall survival or disease-free survival, and postoperative chemotherapy and tumor recurrence had a significant impact on both overall and disease-free survival in stage III patients.

There are, however, some limitations in this study. Firstly, it provides a general analysis of colorectal cancer patients without a detailed analysis of each tumor stage. Secondly, this study does not analyze data regarding the degree of black staining of lymph nodes or the detected number of lymph nodes with a diameter of less than 5 mm, indicating some deficiencies in the analysis of specific indicators. Lastly, we does not count the number of lymph nodes detected outside of the locoregional spread., and we will add data on this aspect in a subsequent study.

In summary, the carbon nanoparticle tracing technique can effectively increase the number of lymph nodes detected during radical resection for colorectal cancer and enhance the accuracy of postoperative pathological N staging, which may assist in guiding adjuvant therapy. However, it has no significant effect on the five-year overall survival rate or disease-free survival rate in patients.

## Data Availability

The original contributions presented in the study are included in the article/supplementary material. Further inquiries can be directed to the corresponding author.

## References

[1] Dekker E Tanis PJ Vleugels JLA Kasi PM Wallace MB. Colorectal cancer. Lancet. (2019) 394:1467–80. doi: 10.1016/S0140-6736(19)32319-0 31631858

[2] Bresalier RS. Colorectal cancer screening in a changing world. Gastroenterol Clinics North America. (2022) 51:577–91. doi: 10.1016/j.gtc.2022.05.002 36153111

[3] Zhang L Cao FC Zhang GY Shi LS Chen SC Zhang ZH. Trends in and predictions of colorectal cancer incidence and mortality in China from 1990 to 2025. Front Oncol. (2019) 9. doi: 10.3389/fonc.2019.00098 PMC6393365 30847304

[4] Sung H Ferlay J Siegel RL Laversanne ML Soerjomataram IS Jemal AJ. Global cancer statistics 2020: GLOBOCAN estimates of incidence and mortality worldwide for 36 cancers in 185 countries. CA: A Cancer J Clin. (2021) 71:209–49. doi: 10.3322/caac.21660 33538338

[5] Qu R Ma YM Zhang ZP Fu WF. Increasing burden of colorectal cancer in China. Lancet Gastroenterol Hepatol. (2022) 7. doi: 10.1016/S2468-1253(22)00156-X 35809603

[6] Xu L Zhao JH Li ZL Sun J Lu Y Zhang RQ. National and subnational incidence, mortality and associated factors of colorectal cancer in China: A systematic analysis and modelling study. J Global Health. (2023) 13. doi: 10.7189/jogh.13.04096 PMC10569376 37824177

[7] Destri GL. Colorectal cancer and lymph nodes: The obsession with the number 12. World J Gastroenterol. (2014) 20. doi: 10.3748/wjg.v20.i8.1951 PMC3934465 24587671

[8] Mounika RN Ananthamurthy A. Lymph node yield in colorectal cancer specimens and its impact on pathological staging: Does number matter? J Cancer Res Ther. (2023) 19:671–4. doi: 10.4103/jcrt.jcrt_980_21 37470592

[9] Resch A. Lymph node staging in colorectal cancer: Old controversies and recent advances. World J Gastroenterol. (2013) 19. doi: 10.3748/wjg.v19.i46.8515 PMC3870496 24379568

[10] Betge J Harbaum LH Pollheimer MJP Lindtner RAL Kornprat PK Ebert MPE. Lymph node retrieval in colorectal cancer: determining factors and prognostic significance. Int J Colorectal Dis. (2017) 32:991–8. doi: 10.1007/s00384-017-2778-8 PMC5486641 28210855

[11] Liu F Peng D Liu X-Y Liu X-R Li Z-W Wei Z-Q. The effect of carbon nanoparticles staining on lymph node tracking in colorectal cancer: A propensity score matching analysis. Front Surg. (2023) 10. doi: 10.3389/fsurg.2023.1113659 PMC10014567 36936663

[12] Koimtzis G Geropoulos G Stefanopoulos L Chalklin CG Karniadakis I Alexandrou V. The role of carbon nanoparticles as lymph node tracers in colorectal cancer: A systematic review and meta-analysis. Int J Mol Sci. (2023) 24. doi: 10.3390/ijms242015293 PMC10607187 37894972

[13] Maffioli A Danelli P. Carbon nanoparticles application during colorectal cancer surgery: Updates from China. Digestive Liver Dis. (2020) 52:1443–4. doi: 10.1016/j.dld.2020.09.023 33097428

[14] Wang Z-H Gang T-R Wu S-S Lu C Gao G-X Xu W. Single-port endoscopic-sentinel lymph node biopsy combined with indocyanine green and carbon nanoparticles in breast cancer. Surg Endoscopy. (2023) 37:7591–9. doi: 10.1007/s00464-023-10018-9 PMC10520094 37460818

[15] Zhang L Huang Y Yang C Zhu T Lin Y Gao H. Application of a carbon nanoparticle suspension for sentinel lymph node mapping in patients with early breast cancer: a retrospective cohort study. World J Surg Oncol. (2018) 16. doi: 10.1186/s12957-018-1414-6 PMC6006710 29914538

[16] Lei Y Zhao Z-M Li Y-S. Assessment of the efficacy and safety of carbon nanoparticles-guided lymph node dissection in gastric cancer surgery: a systematic review and meta-analysis. Int J Clin Oncol. (2023) 28:764–76. doi: 10.1007/s10147-023-02333-x 37099219

[17] Feng Y Yang K Sun H-H Liu Y-P Zhang D Zhao Y. Value of preoperative gastroscopic carbon nanoparticles labeling in patients undergoing laparoscopic radical gastric cancer surgery. Surg Oncol. (2021) 38. doi: 10.1016/j.suronc.2021.101628 34174770

[18] Liu P Tan J Tan Q Xu L He T Lv Q. Application of carbon nanoparticles in tracing lymph nodes and locating tumors in colorectal cancer: A concise review. Int J Nanomed. (2020) 15:9671–81. doi: 10.2147/IJN.S281914 PMC7719328 33293812

[19] Li X. The safety and effectiveness of carbon nanoparticles suspension in tracking lymph node metastases of colorectal cancer: a prospective randomized controlled trial. Japanese J Clin Oncol. (2020) 50:535–42. doi: 10.1093/jjco/hyaa011 32083298

[20] Saha S Phelimon B Efeson M Helina A Elgamal M Kiya G. The role of sentinel lymph node mapping in colon cancer: detection of micro-metastasis, effect on survival, and driver of a paradigm shift in extent of colon resection. Clin Exp Metastasis. (2021) 39:109–15. doi: 10.1007/s10585-021-10121-y 34698993

[21] Ichimasa K Kudo S-E Miyachi H Kouyama Y Mochizuki K Takashina Y. Current problems and perspectives of pathological risk factors for lymph node metastasis in T1 colorectal cancer: Systematic review. Digestive Endoscopy. (2022) 34:901–12. doi: 10.1111/den.14220 34942683

[22] Chang GJ Rodrigas-Bigas MA Skibber JM Moyer VA. Lymph node evaluation and survival after curative resection of colon cancer: systematic review. JNCI J Natl Cancer Institute. (2007) 99:433–41. doi: 10.1093/jnci/djk092 17374833

[23] Sato K Shimoda H Miura T Sakamoto Y Morohashi H Watanabe S. Widespread anorectal lymphovascular networks and tissue drainage: analyses from submucosal India ink injection and indocyanine green fluorescence imaging. Colorectal Dis. (2021) 23:1334–45. doi: 10.1111/codi.15582 PMC8248146 33570769

[24] Carvalho A Gonçalves N Teixeira P Goulart A Leão P. The impact of methylene blue in colorectal cancer: Systematic review and meta-analysis study. Surg Oncol. (2024) 53. doi: 10.1016/j.suronc.2024.102046 38377643

[25] Emile SH Elfeki H Shalaby M Sakr A Sileri P Laurberg S. Sensitivity and specificity of indocyanine green near-infrared fluorescence imaging in detection of metastatic lymph nodes in colorectal cancer: Systematic review and meta-analysis. J Surg Oncol. (2017) 116:730–40. doi: 10.1002/jso.v116.6 28570748

[26] Park JW Sohn DK Hong CW Han KS Choi DH Chang HJ. The usefulness of preoperative colonoscopic tattooing using a saline test injection method with prepackaged sterile India ink for localization in laparoscopic colorectal surgery. Surg Endoscopy. (2007) 22:501–5. doi: 10.1007/s00464-007-9495-2 17704874

[27] Nagata J Fukunaga Y Akiyoshi T Konishi T Fujimoto Y Nagayama S. Colonic marking with near-infrared, light-emitting, diode-activated indocyanine green for laparoscopic colorectal surgery. Dis Colon Rectum. (2016) 59:e14–8. doi: 10.1097/DCR.0000000000000542 26734978

[28] Yan J Xue F Chen H Wu X Zhang H Chen G. A multi-center study of using carbon nanoparticles to track lymph node metastasis in T1–2 colorectal cancer. Surg Endoscopy. (2014) 28:3315–21. doi: 10.1007/s00464-014-3608-5 24935202

[29] Wang D Chen M Lv L Chen Y Tian K Chairat S. Injection of carbon nanoparticles for lymph node detection after laparoscopic colorectal cancer surgery. J Nanosci Nanotechnol. (2021) 21:886–94. doi: 10.1166/jnn.2021.18667 33183420

[30] Wang L-Y Li J Zhou X Zheng Q-C Cheng X. Clinical application of carbon nanoparticles in curative resection for colorectal carcinoma. OncoTargets Ther. (2017) 10:5585–9. doi: 10.2147/OTT.S146627 PMC5702162 29200873

[31] Cai JW Li XL Chen X Rong YM Tan YX Weng JR. [Application of carbon nanoparticles mapping lymph nodes in curative resection for colorectal carcinoma]. Zhonghua Wei Chang Wai Ke Za Zhi. (2020) 23:990–5. doi: 10.3760/cma.j.cn.441530-20200728-00447 (Chinese). 33053995

[32] Wang R Zhan HL Li DZ Li HT Yu L Wang W. [Application of endoscopic tattooing with carbon nanoparticlet in the treatment for advanced colorectal cancer]. Zhonghua Wei Chang Wai Ke Za Zhi. (2020) 23:56–64. doi: 10.3760/cma.j.issn.1671-0274.2020.01.010 (Chinese). 31958932

